# ASSOCIATION OF SECOND GENERATION EPIGENETIC CLOCKS AND DISCRIMINATION WITH TRAJECTORIES OF CHRONIC HEALTH CONDITIONS

**DOI:** 10.1093/geroni/igad104.0180

**Published:** 2023-12-21

**Authors:** Miriam Mutambudzi, Maria Brown, Nai-Wei Chen

**Affiliations:** Syracuse University, Syracuse, New York, United States; Syracuse University, Syracuse, New York, United States; University of Missouri, Columbia, Missouri, United States

## Abstract

This study aimed to examine whether 2nd generation epigenetic clocks and perceived discrimination as measured by the Everyday Discrimination Scale are associated with differential classification into trajectories of chronic health conditions (CHCs). We used 2014-2020 data from the Health and Retirement Study (HRS) and biological clock data from the 2016 HRS Venous Blood Study (N=3,177). CHC trajectories were constructed using latent class growth models. Multinomial logistic regression analysis examined the association between PhenoAge and GrimAge epigenetic clocks respectively, perceived discrimination, and the constructed trajectories. Participants (≥ 50 years) were classified into 4 CHC trajectory classes, graded by risk of increasing mean CHCs, with trajectory class 1 indicating the lowest mean number of CHCs (most favorable) and class 4 the highest (worst), over 3 study waves. Approximately 35.5% Black, 24.9% Hispanic, and 25% White participants reported high discrimination (p< 0.01). Compared to Whites, Blacks had increased risk of classification into trajectory class 4 (RRR=1.91, p=0.02 GrimAge and RRR=2.31, p< 0.01 PhenoAge). PhenoAge was associated with increased risk of classification into trajectory class 4 for Hispanics (RRR=1.17, 95%CI=1.06-1.28), and modest increases in risk for Blacks (RRR=1.07, 95%CI=1.01-1.15) and Whites (RRR=1.06, 95%CI=1.03-1.09). GrimAge was associated with increased risk of classification into trajectory class 4 for Hispanics (RRR=1.21, 95%CI=1.06-1.38), Blacks (RRR=1.19, 95%CI=1.06-1.33) and Whites (RRR=1.14, 95%CI=1.08-1.20). Despite higher observed rates of discrimination in Blacks, experiences of discrimination increased risk of classification into trajectory class 4 for whites only (RRR=1.59, 95%CI=1.06-2.38). Discrimination findings should be interpreted with caution and future work should account for coping strategies.

